# Regional Variation in Skull Thickness Along a Horizontal Plane: Implications for Neuroanatomy Teaching, Neurosurgery, Radiology and Forensic Analysis

**DOI:** 10.7759/cureus.98489

**Published:** 2025-12-04

**Authors:** Chandan Lal Gupta, Shyamalendu Medda, Subhash Bhukya, Paramita Mukhopadhyay, Anirban Das Gupta, Biswabina Ray, Vineet Kumar Kamal

**Affiliations:** 1 Anatomy, All India Institute of Medical Sciences, Kalyani, Kalyani, IND; 2 Anatomy, Institute of Post Graduate Medical Education and Research, Kolkata, IND; 3 Anatomy, Raiganj Government Medical College and Hospital, Raiganj, IND; 4 Biostatistics, All India Institute of Medical Sciences, Kalyani, Kalyani, IND

**Keywords:** burr hole craniotomy, cranium sectioning, ct scan head, forensic anthropology, neuroanatomy, skull thickness

## Abstract

Objective

To quantify skull thickness variation at a horizontal plane passing 2 cm above the highest points of the left and right supraorbital margins anteriorly and the external occipital protuberance posteriorly and to propose a standardized six-region classification for cranial margins.

Methods

Fifteen adult dry basilar skulls from four publicly funded medical institutions in Eastern India were studied. The skull margin at the defined plane was marked at 1-cm intervals between the anterior and posterior midlines on both sides. Thickness was measured using a digital micrometer by three independent observers, with mean values used for analysis. The margin was divided into six regions per side: anteromedial (AM), anterolateral (AL), lateral anterior (LA), lateral posterior (LP), posterolateral (PL), and posteromedial (PM). Inter-observer reliability was assessed using a two-way random-effects intraclass correlation coefficient (ICC). All statistical analyses were performed using Stata v18.

Results

A total of 2,235 measurements were analyzed. Thickness decreased from AM to LA regions and then increased toward the PM region. The LA region was thinnest (left=3.144±0.286 mm; right=3.234±0.809 mm), while the PM region was thickest (left=10.638±1.661 mm; right=11.266±1.659 mm). ICC exceeded 0.9 for over 80% of measurements, confirming high measurement consistency.

Conclusions

This study establishes a reproducible six-region classification and provides precise morphometric data along a clinically important horizontal plane. Apart from its use in cranium sectioning in cadavers, the findings can guide safe neurosurgical entry points, improve radiological lesion localization, and support forensic cranial analysis.

## Introduction

The anatomical exploration of the human brain and its protective meninges is a fundamental aspect of medical education within the field of neuroanatomy. Traditionally, the exposure of these structures in cadavers has been achieved through a specific transverse sectioning of the skull, following a cruciate incision of the scalp and reflection of the four resultant flaps to their respective quadrants (left anterior, right anterior, right posterior, and left posterior). The precise plane for cranium sectioning has been debated and varies according to different dissection manuals. Cunningham’s manual suggests a plane not exceeding 1 cm above the orbital margins and external occipital protuberance [1], while *Grant’s Dissector* advocates a plane 2 cm superior to the supraorbital margin, extending posteriorly across the external occipital protuberance [2]. The technique involves the use of a manual saw to cut through the outer table and diploe, followed by the application of a chisel and mallet to break the inner table. Although this practice has been widely used across medical colleges in India and South-East Asia, it presents four major concerns (encountered during our own practice) as follows: The manual sawing of the hard outer table is laborious and time-intensive. The use of a chisel and mallet to break the inner table creates bone chips and fragments, posing a risk of traumatic injury to fingers when detaching the dura from the cranial fossa and during brain extraction. There is a risk of inadvertent damage to the dural venous sinuses, particularly the transverse sinus and confluence of sinuses, and to the brain due to subjective differences in the pressure applied while hitting the inner table. Sectioning at the lower plane [1] compromises the integrity of internal cranial structures, namely the orbital roof, lateral parts of lesser wing of sphenoid, and the superior petrosal border.

Since the aforementioned manuals provide no information on skull thickness or the depth of the cut, which is critical for avoiding damage to the underlying meninges/brain, we referred to the literature in neurosurgery where halo pin fixation is used to immobilize the cervical spine in trauma. The halo pin is screwed into the calvaria to a particular depth, safeguarding the meninges. The data related to the studies on halo pin placement were gathered [3-6].

The existing references do not solve the issue of sectioning the cranium as far as anatomical dissection is concerned. To overcome the limitations of the traditional method, based on our dissection experience, we propose an alternative approach that involves sectioning at a higher plane using a motorized circular saw, which could minimize damage to important intracranial structures (mentioned above) and prevent the generation of bony fragments. However, a lack of knowledge regarding the precise thickness of the skull in this new plane could potentially damage the meninges and brain. Therefore, the present study was aimed to fill this gap by measuring the thickness of dry skulls at small and regular intervals along the circumference at a plane 2 cm superior to the highest point on supraorbital margin (of both left and right sides) anteriorly, and 2 cm superior to the most prominent point on the external occipital protuberance posteriorly.

## Materials and methods

The data related to the studies on halo pin placement were gathered. The findings are summarized in Table 1 [3-6].

**Table 1:** Comparative analysis of the thickness of the skull margin It shows a comparative analysis of the thickness of the skull margin and the plane of reference, as described in selected previous literature.

Sl no.	Study population /subject	Thickness	Plane of reference	Limitation	Reference
1	CT scan films of 270 children and adolescents ranging from less than 6 months to 17 years age	Minimum mean thickness of 1.97±0.44 mm in left lateral region in children less than 4 years, and a maximum mean thickness of 8.63±2.31 mm in posterior region in adolescents aged above 13 years	Above the eyebrows and lateral to supraorbital nerves (anterolateral region), and posterior to ear above auditory meatus	No mention about the exact level at which the thickness is measured. For the dissection and teaching of medical students, mostly the older adults and elderly cadavers (donated bodies) are used. Adolescent skulls are skeletally immature and thinner and hence the thickness mentioned in this work does not hold true for skeletally mature skull of the adults.	[3]
2	21 elderly cadaveric skulls	Average thickness of 7.36±1.57 mm in the anterolateral region and 9.47±1.12 mm in the posterolateral region	Anterolateral region was 10 mm superior to orbital rim at the junction of medial 2/3rd and lateral 1/3rd of supraorbital margin. The posterolateral region was at 4 o’clock and 8 o’clock positions considering direct anterior midline at 12 o’clock position	Exact level at which the thickness in the posterolateral region was measured has not been mentioned. Moreover, this data is not sufficient in order to approach the removal of calvaria.	[4,5]
3	CT scans of heads of 415 individuals (on a wide range of age from 15 days till 89 years)	5.62 to 7.42 mm in the anterior midline, 5.52 to 8.54 mm in the anterolateral and 5.59 to 8.86 mm in the posterolateral regions	Anterolateral thickness was measured at 1 cm above orbital rim, and superior to lateral 2/3^rd^ of orbit. The posterolateral thickness was measured at 1 cm above and 1 cm behind the superior edge of the pinna.	No mention of the thickness along the entire circumference and this data too is insufficient in order to approach removal of calvaria.	[6]

Selection of the skulls

A total of 31 basilar skulls were collected from the Department of Anatomy of four publicly funded medical institutions in and around Kolkata (India) and 15 of them were selected for the study. The selected skulls were labelled with a four-digit alphanumeric code (AA-XX), where AA represents the institute and XX the serial number of the skull. The remaining 16 basilar skulls were excluded due to one or more of the following reasons: (1) the transverse margin of the skull was broken, making the continuous placement of points at uniform distances impossible; (2) the plane of transverse sectioning of the skull was much lower than the desired plane, resulting in some points being omitted at the plane of measurement; (3) the plane of transverse sectioning of the skull was much higher than the desired plane, making the measuring tool difficult to position precisely at the required points; (4) skulls with one or more sutures not fused, thereby excluding the skulls from adolescents and early adults. The exact or approximate age and gender of the skulls could not be retrieved from the respective institution, despite all possible efforts, and could not be ascertained due to funding limitations.

Marking the desired plane

**Figure 1:**
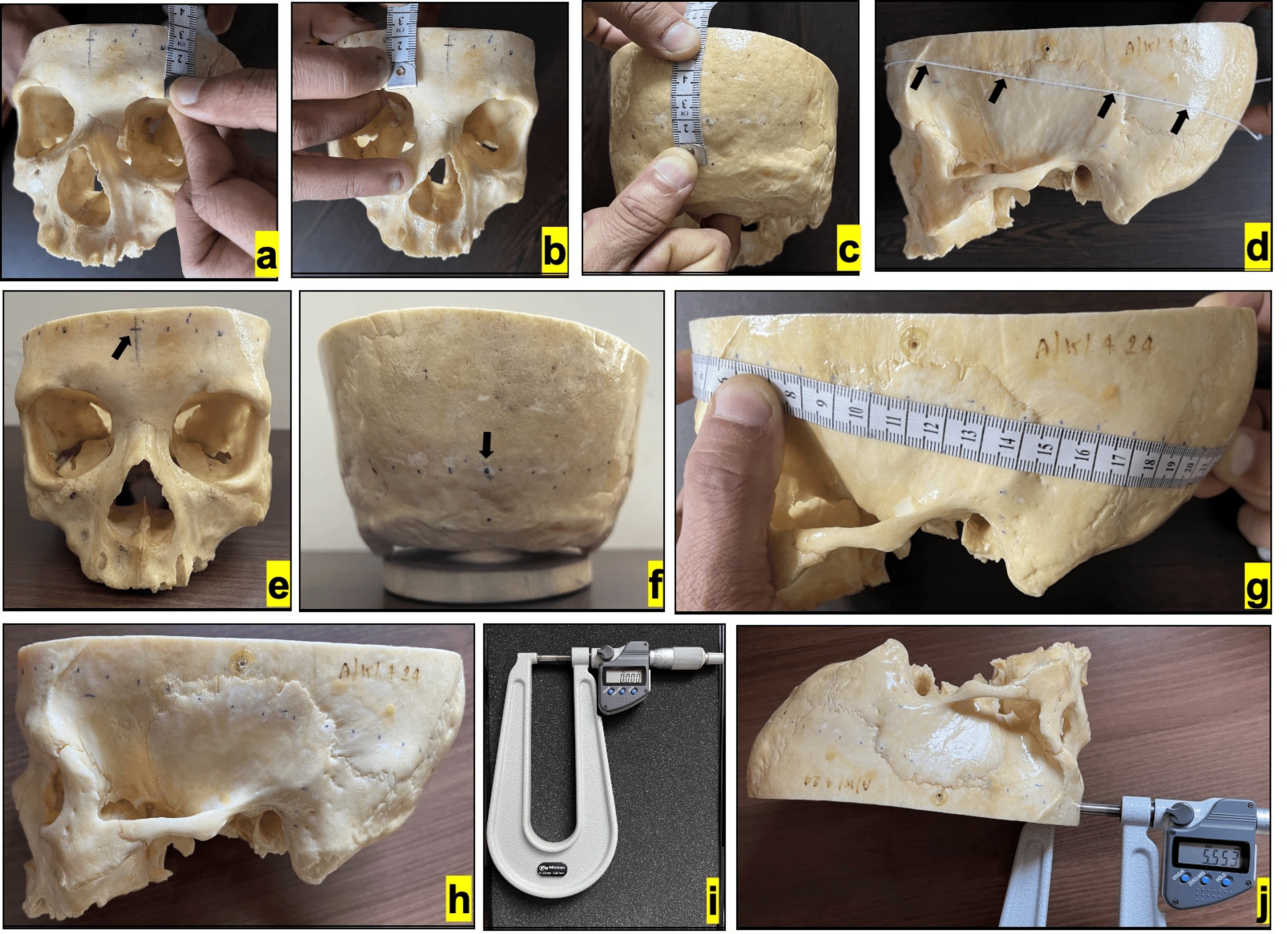
Steps of skull measurement Representative photographs (captured after the actual measurement) showing the method of measurement. (a, b) Localizing the point 2 cm above the highest point of supraorbital margin on the left and right sides. (c) Localizing the point 2 cm above the most prominent point on the external occipital protuberance. (d) An inelastic thread tied at the plane passing through a, b, and c to measure the circumference. (e and f) Localizing the anterior midline (AM) and posterior midline (PM) (arrow marked). (g, h) Localizing the points at 1 cm intervals starting from AM. (i) The digital micrometer used in the study. The process of measuring thickness (j).

To demarcate the specific anatomical plane, the following procedure was adopted: Three points were marked on the external surface of the basilar skull: 2 cm superior to the highest point of the supraorbital margin on both the left and right sides, and 2 cm superior to the most prominent point on the external occipital protuberance. An inelastic thread was fastened around the circumference in such a way that it passes through the three points (Figure 1a-d).

With the thread in place, a line (line 1) was traced on the skull, which represents the desired anatomical plane. Subsequently, the thread was removed, and its length was measured to determine the circumference of the skull at the marked plane. The values were noted in centimeters (cm, up to 1 decimal place).

Marking the midlines

The anterior midline was established by drawing a vertical line (line 2) from the midpoint of frontonasal suture upwards, intersecting line 1 (Figure 1e). The posterior midline was identified as a point located 2 cm above the most prominent part of the external occipital protuberance (Figure 1f).

Marking the interval points

Starting from the anterior midline, points were marked at 1 cm intervals along line 1 on both the left and right sides of the skull using a tailor's tape (Figure 1g, h). Line 1, line 2, midlines and interval points were drawn in the presence and concordance of three authors to avoid inter-observer bias. Line 1 was erased after marking the interval points.

Measurement of skull thickness

The thickness of the skull (i.e. from the external surface of the outer table to the internal surface of the inner table) was measured using a digital sheet metal micrometer (Mitutoyo PMUS150-25MX, product code: 389-251-30; Mitutoyo Corporation, Kawasaki-shi, Japan) (Figure 1i, j). The micrometer used in the study has an accuracy of ±4 µm, a resolution = 0.001 mm, flatness=0.6 µm, and parallelism of ≤3 µm. Since basilar skulls were used for measurement, the method of contacting the inner table was therefore directly visible to human eyes. To minimize human error, three observers independently measured the thickness at each point, and the average of the three was considered for statistical analysis. The dryness of the skulls and carbide-tipped opposing surfaces of themicrometer ensured zero compressibility. The thickness values were recorded in millimetres (mm, up to three decimal places). On a successful completion of the measurements, all labels and marks (points) were erased from the skulls and then returned to their respective institutions.

Defining the regions

**Figure 2:**
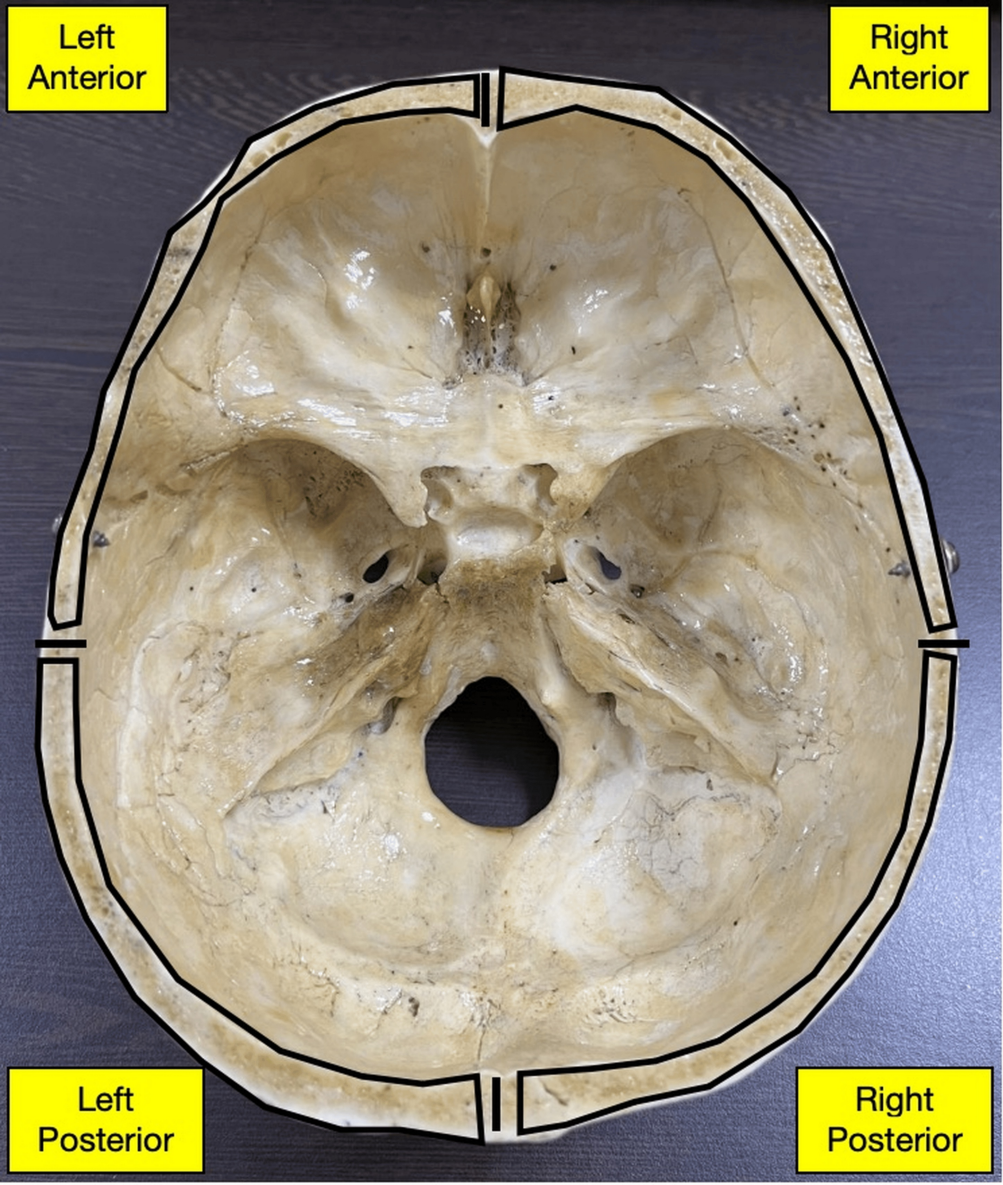
Four quadrants in the skull margin Four quadrants (left anterior, left posterior, right anterior, and right posterior) in the skull margin

The number of points (at 1 cm interval) on the left and right sides of the skull between the anterior and posterior midlines varied between 22 and 26. Out of the 15 skulls, seven had an equal number of points on both the right and left sides (five skulls with 24 points and two skulls with 23 points on each side). Among the remaining eight asymmetrical skulls, five had one extra point on the right side ((24,23); (24,23); (24,23); (23,22); (26,25)) and three had one extra point on the left side ((23,24); (22,23); (24,25)). Since no regular or definite pattern was observed in 15 skulls with respect to left and right-sided symmetry or asymmetry, and since there was no evidence found in the literature with respect to definite criteria on how to divide the margin of the skull into different regions, the following mathematical principle was adopted. For an even number of points (n), the imaginary point between (n/2)th point and ((n/2)+1)th point was considered the middle point for that side. For an odd number of points, ((n+1)/2)th point was considered the midpoint for that side. With respect to the midpoint of a particular side (left/right), the anterior half and posterior half were described. Thus, in each basilar skull, four quadrants were defined along the margin: left anterior, left posterior, right anterior, and right posterior (Figure 2).

**Figure 3:**
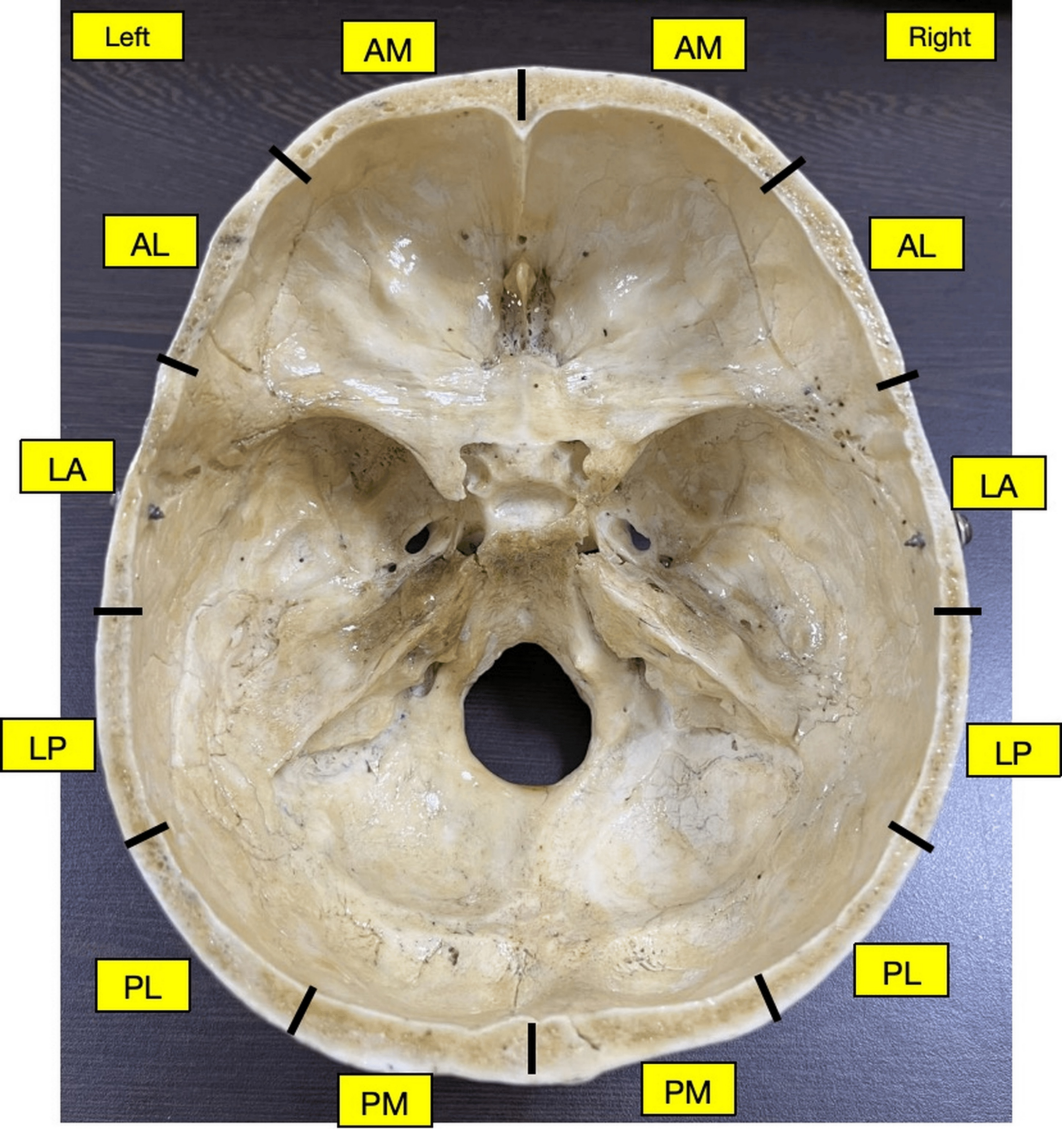
Six regions of the skull margin Proposed six regions of the skull margin on the left and right sides. AM: Anteromedial; AL: anterolateral; LA: lateral anterior; LP: lateral posterior; PL: posterolateral; PM: posteromedial.

Each quadrant was further subdivided into three regions. In the anterior quadrants, anteromedial (AM), anterolateral (AL), and lateral anterior (LA) regions, and in posterior quadrants, Lateral posterior (LP), posterolateral (PL), and posteromedial (PM) regions were defined in sequence. Thus, each half margin was divided into six regions, namely AM, AL, LA, LP, PL, PM in sequence from anterior to posterior (Figure 3).

To distribute the points among these six regions, the following method was followed.

In the case of 12 points in any quadrant, four points were considered in each region in sequence. In the case of 11 points in anterior/posterior quadrant, three points were considered in LA/LP region, respectively; in the same way, in the case of 13 points in anterior/posterior quadrant, five points were considered in LA/LP region, respectively. The remaining regions in that quadrant contained four points each. This method of distributing points was adopted due to the relative flatness, thinness, and lesser variability of the LA/LP region compared to other regions. It formed the basis for calculating the average thickness in each of the six regions in each half of the skull margin. The mean thickness for the AM, AL, PL, and PM regions in each skull was calculated by taking the average thickness of four points in that region. For LA and LP regions in each skull, the mean thickness was calculated by taking the average thickness of 3/4/5 points in that region (as the case for that skull).

Statistical analysis

The quantitative values of regional thickness were presented as mean±standard deviation. Pearson correlation coefficients were used to assess the relationship between circumference and midline thickness, as well as regional thicknesses on the left and right sides. Error bar graphs were plotted based on the mean and standard deviation of the thickness measurements at each region on the left and right sides of the skull. Additionally, a line graph depicting the coefficient of variation (CV) was generated for six areas along the left and right skull margins to illustrate the variability in regional thickness. All statistical analyses were performed using Stata v. 18 (StataCorp LLC, College Station, TX, USA). Inter-observer reliability was quantified using the intraclass correlation coefficient (ICC). We used a two-way random-effects model with absolute agreement, commonly referred to as ICC. It checks how consistently three independent observers measured skull thickness at the same points. ICC values close to one indicate excellent agreement. A 95% confidence interval for the ICC was computed using the standard F-distribution method to show the precision of these reliability estimates. Normality of continuous variables (regional thickness values) was examined using the Shapiro-Wilk test and by visual inspection of histograms. As the variables exhibited approximate normal distribution, Pearson correlation coefficients were deemed appropriate. To avoid analytical inflation due to multiple measurements per skull, all correlation and descriptive analyses were conducted using the region-wise mean thickness values for each skull, rather than point-level data. Thus, each skull contributed one value per region, mitigating issues related to clustering.

## Results

A total of 2235 measurements were made on 15 skulls, which include circumference, thickness at midlines, and thickness at 1 cm interval between the midlines along the left and right sides of the skull margin.

Circumference and midline thickness

The external circumference of the skulls ranged between 45.8 cm and 51.7 cm. No regular/ definite pattern was seen with regard to the thickness at the anterior and posterior midlines. The skull with the smallest circumference (i.e. 45.8 cm) showed nearly equal thickness at the anterior (11.103 mm) and posterior (11.620 mm) midlines. The skull with the greatest circumference (i.e. 51.7 cm) showed greater thickness at the anterior (10.444 mm) than at the posterior (8.640 mm) midline. The skull with an intermediate circumference value (i.e. 48.6 cm) showed greater thickness at the anterior (9.205 mm) than at the posterior (6.097 mm) midline. Thus, the skull with the smallest circumference showed greater thickness at both the anterior and posterior midlines than the skull with the greatest circumference. Out of the 15 skulls, the anterior midline was thicker than the posterior midline in eight skulls, and vice versa in the remaining seven. The difference ranged from 1.803 mm to 7.868 mm in the former and 0.517 mm to 4.501 mm in the latter case (Table 2).

Mean regional thickness

**Figure 4:**
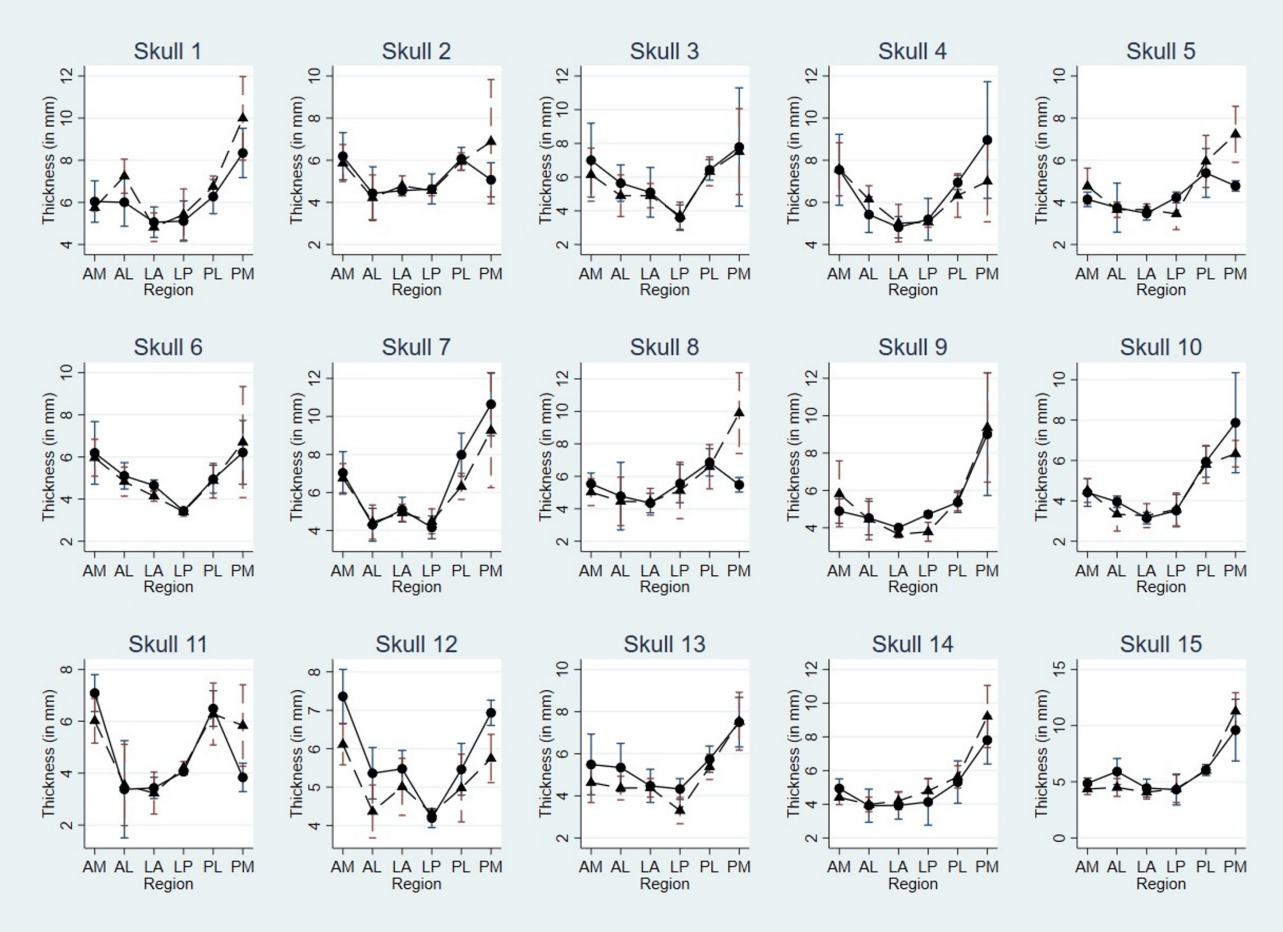
Thickness (mean and SD) in the six regions on left and right sides of skull margin The figure shows thickness (mean and SD) in the six regions on left and right sides of skull margin in all 15 skulls. “circle” and “triangle” represent values (expressed in mm) on the left and right sides, respectively. In the majority of the skulls, the posteromedial (PM) region shows maximum asymmetry.

The mean thickness in six regions on the left and right sides of the skull margin in all 15 skulls is shown in Figure 4.

**Figure 5:**
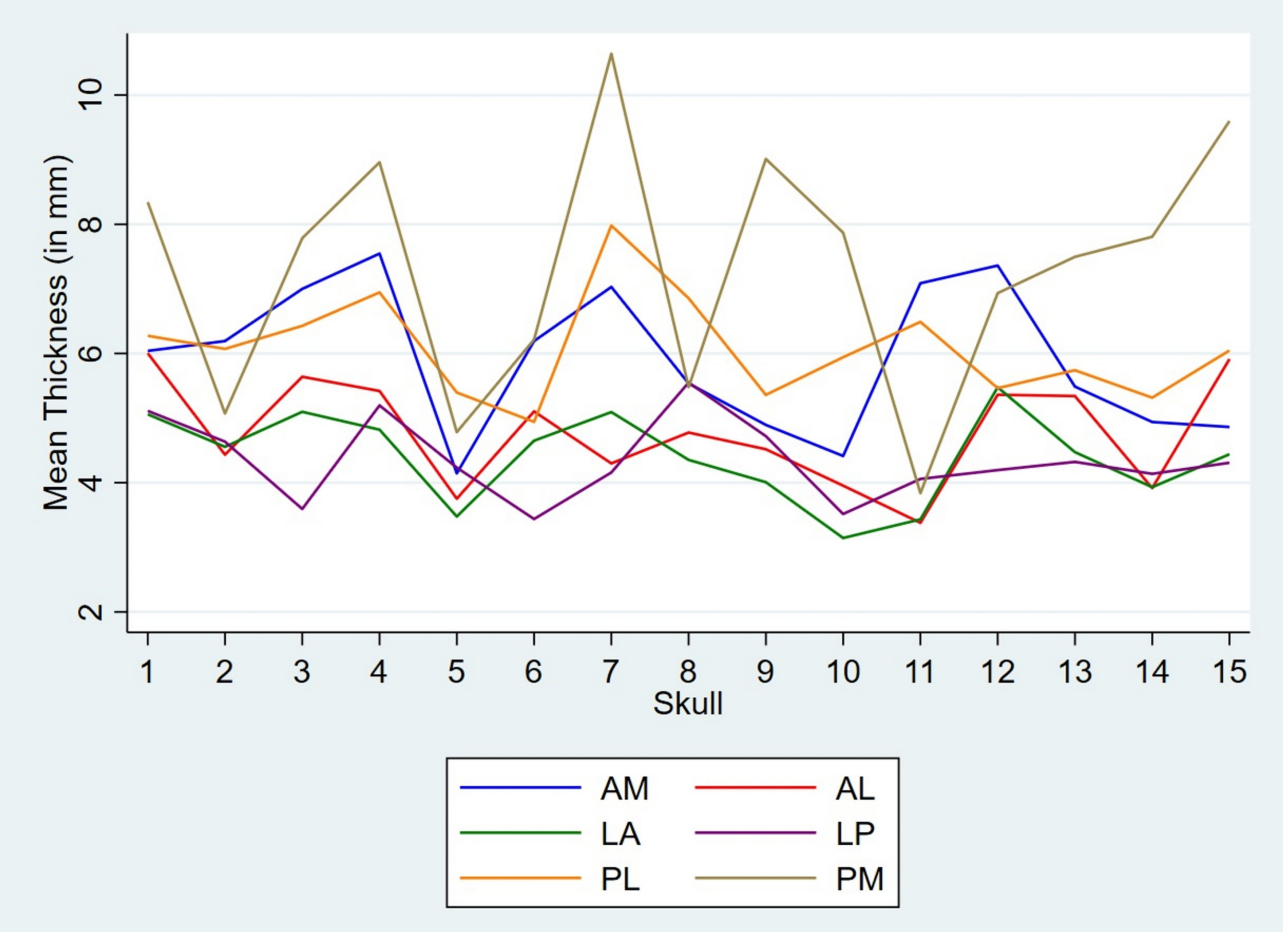
Variation of mean thickness of six regions on the left side The figure shows variation of mean thickness of six regions on the left side among all 15 skulls. Nearly 50% of the skulls show minimum thickness in the LA region. The PM region shows maximum thickness in the majority of the skulls.

**Figure 6:**
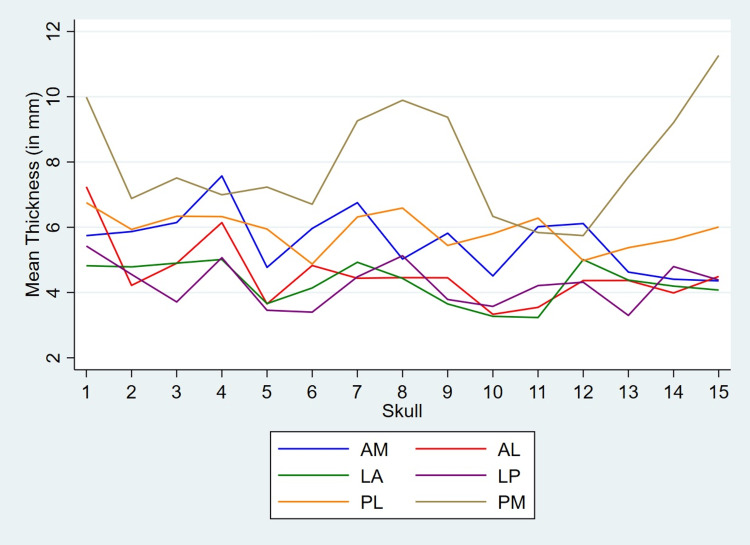
Variation of mean thickness of six regions on the right side The figure shows variation of mean thickness of six regions on the right side among all 15 skulls. Nearly 50% of the skulls show minimum thickness in the LP region. The PM region shows maximum thickness in the majority of the skulls.

In majority of the skulls (11 out of 15), PM region shows maximum asymmetry. LA and LP regions show minimum variability in thickness. The variation of mean thickness of six regions on left and right sides in all 15 skulls are shown in Figures 5, 6, respectively.

**Figure 7:**
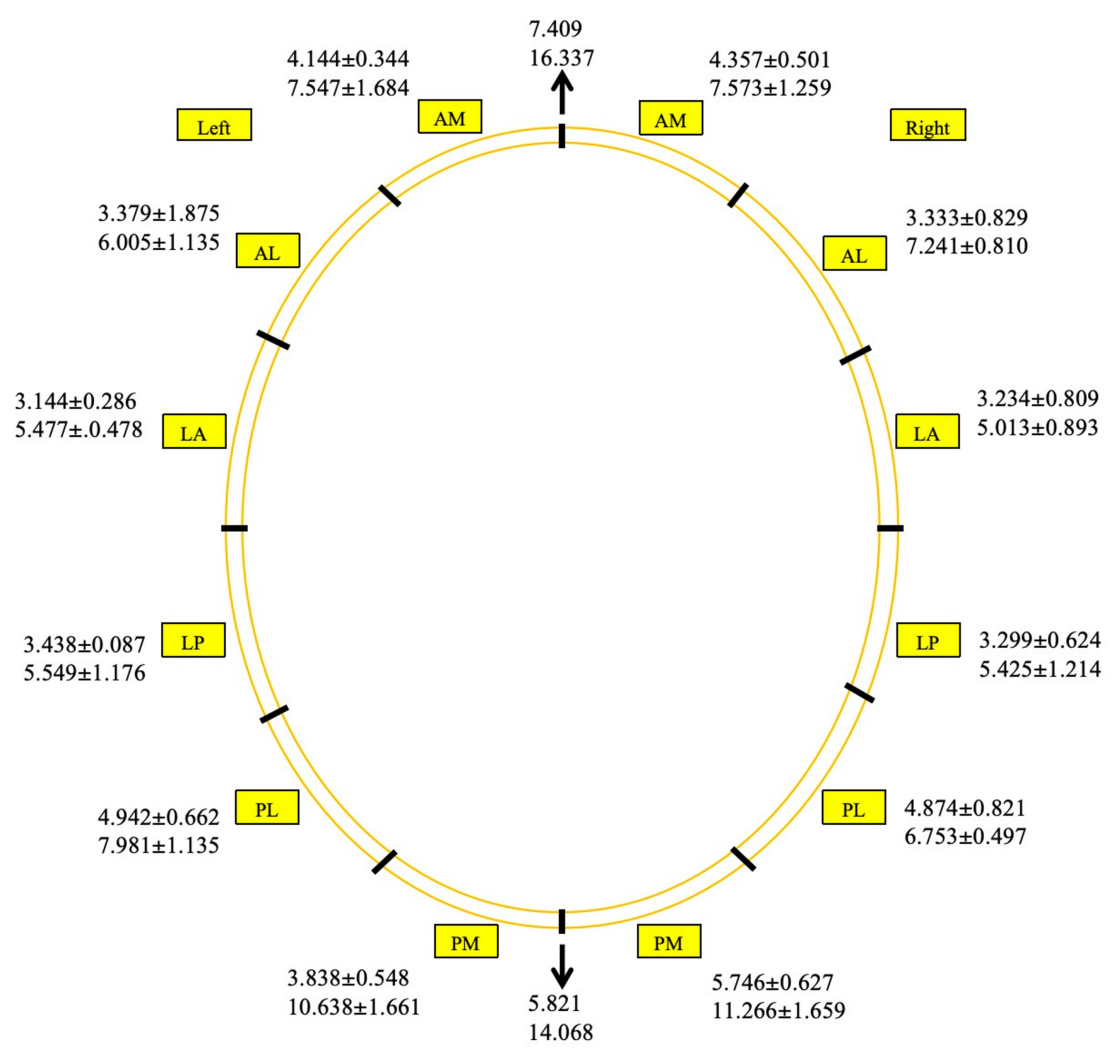
Minimum and maximum mean thickness (in mm) at midlines and in the six regions Minimum and maximum mean thickness (in mm) at anterior and posterior midlines and in the six regions on each side along the margin of skull. Midline thickness values are expressed as “mean” and regional thickness values are expressed as “mean±SD”. AM: Anteromedial; AL: anterolateral; LA: lateral anterior; LP: lateral posterior; PL: posterolateral; PM: posteromedial.

The minimum and maximum mean thickness in each region is shown in Figure 7 and Table 2.

**Table 2:** Circumference and thickness at midlines and six regions It shows the values of circumference and thickness at the anterior midline, posterior midline, and six regions on each left and right sides in the 15 skulls. The circumference value is expressed in centimeters (cm) up to 1 decimal, and the remaining values are expressed in millimeters (mm) up to 3 decimals. Midline thickness values are expressed as mean and regional thickness values are expressed as “mean±SD”. AM: Anteromedial; AL: anterolateral; LA: lateral anterior; LP: lateral posterior; PL: posterolateral; PM: posteromedial.

Serial no of skull	Circumference (in cm)	Anterior Midline	Posterior Midline	Side	AM	AL	LA	LP	PL	PM
1	48.5	10.095	12.680	L	6.04±0.983	6.005±1.135	5.059±0.724	5.112±0.956	6.274±0.814	8.344±1.171
				R	5.744±0.148	7.241±0.81	4.821±0.68	5.425±1.214	6.753±0.497	9.99±1.985
2	49	16.337	8.469	L	6.193±1.117	4.433±1.255	4.555±0.236	4.636±0.712	6.071±0.537	5.071±0.811
				R	5.868±0.877	4.219±1.083	4.786±0.472	4.557±0.244	5.934±0.419	6.882±2.948
3	49.1	14.424	12.621	L	7±2.194	5.64±1.082	5.098±1.475	3.594±0.754	6.428±0.611	7.785±3.508
				R	6.145±1.575	4.897±1.236	4.901±0.718	3.712±0.801	6.337±0.854	7.512±2.547
4	48.5	15.548	12.510	L	7.547±1.684	5.419±0.85	4.821±0.506	5.196±0.993	6.945±0.341	8.96±2.767
				R	7.573±1.259	6.143±0.648	5.013±0.893	5.068±0.247	6.327±1.033	6.996±1.917
5	48.6	9.205	6.097	L	4.144±0.344	3.752±1.165	3.476±0.317	4.234±0.255	5.396±1.157	4.782±0.245
				R	4.771±0.853	3.651±0.364	3.661±0.265	3.456±0.749	5.946±1.235	7.231±1.332
6	45.8	11.103	11.620	L	6.192±1.485	5.103±0.624	4.65±0.261	3.438±0.087	4.942±0.662	6.216±1.511
				R	5.964±0.876	4.828±0.69	4.14±0.241	3.399±0.209	4.874±0.821	6.705±2.636
7	50.5	9.720	14.068	L	7.03±1.114	4.299±0.865	5.093±0.646	4.158±0.594	7.981±1.135	10.638±1.661
				R	6.754±0.767	4.438±0.898	4.928±0.452	4.482±0.654	6.315±0.685	9.261±3.01
8	51.7	10.444	8.640	L	5.536±0.66	4.776±2.081	4.353±0.6	5.549±1.176	6.856±0.844	5.479±0.448
				R	5.026±0.832	4.456±1.489	4.433±0.821	5.127±1.74	6.589±1.362	9.892±2.489
9	47.8	11.616	13.720	L	4.894±0.65	4.518±0.898	4.008±0.12	4.72±0.165	5.359±0.536	9.011±3.284
				R	5.818±1.762	4.452±1.095	3.65±0.182	3.787±0.507	5.444±0.539	9.371±2.929
10	49.3	13.699	9.815	L	4.414±0.685	3.954±0.283	3.144±0.286	3.516±0.799	5.942±0.772	7.87±2.477
				R	4.508±0.585	3.333±0.829	3.271±0.592	3.576±0.815	5.804±0.935	6.333±0.658
11	48.3	13.520	5.821	L	7.087±0.712	3.379±1.875	3.432±0.409	4.058±0.123	6.489±0.686	3.838±0.548
				R	6.017±0.86	3.546±1.567	3.234±0.809	4.214±0.234	6.281±1.195	5.838±1.569
12	48	10.064	6.655	L	7.361±0.702	5.362±0.669	5.477±0.478	4.195±0.248	5.464±0.675	6.934±0.329
				R	6.114±0.533	4.367±0.689	5.009±0.741	4.316±0.08	4.977±0.881	5.746±0.627
13	50	10.909	13.933	L	5.488±1.438	5.342±1.145	4.473±0.787	4.322±0.495	5.741±0.621	7.496±1.176
				R	4.627±0.946	4.369±0.56	4.381±0.443	3.299±0.624	5.375±0.599	7.541±1.373
14	50	7.409	11.910	L	4.94±0.566	3.916±0.983	3.931±0.815	4.138±1.378	5.316±1.257	7.806±1.419
				R	4.407±0.432	3.988±0.434	4.197±0.521	4.796±0.731	5.623±0.665	9.206±1.846
15	46.5	10.016	13.342	L	4.862±0.473	5.914±1.159	4.44±0.798	4.309±1.372	6.047±0.484	9.598±2.752
				R	4.357±0.501	4.493±0.795	4.076±0.615	4.384±1.226	6.008±0.342	11.266±1.659

The minimum mean thickness was seen in the LA region (left=3.144±0.286 mm, right=3.234±0.809 mm). The maximum mean thickness was found in the PM region (left=10.638±1.661 mm, right=11.266±1.659 mm). The AM, LA, and LP regions in the left and right sides showed similar minimum and maximum mean thickness. The PM region showed great variation and a wide range in minimum and maximum mean thickness.

Coefficient of variation in thickness

**Figure 8:**
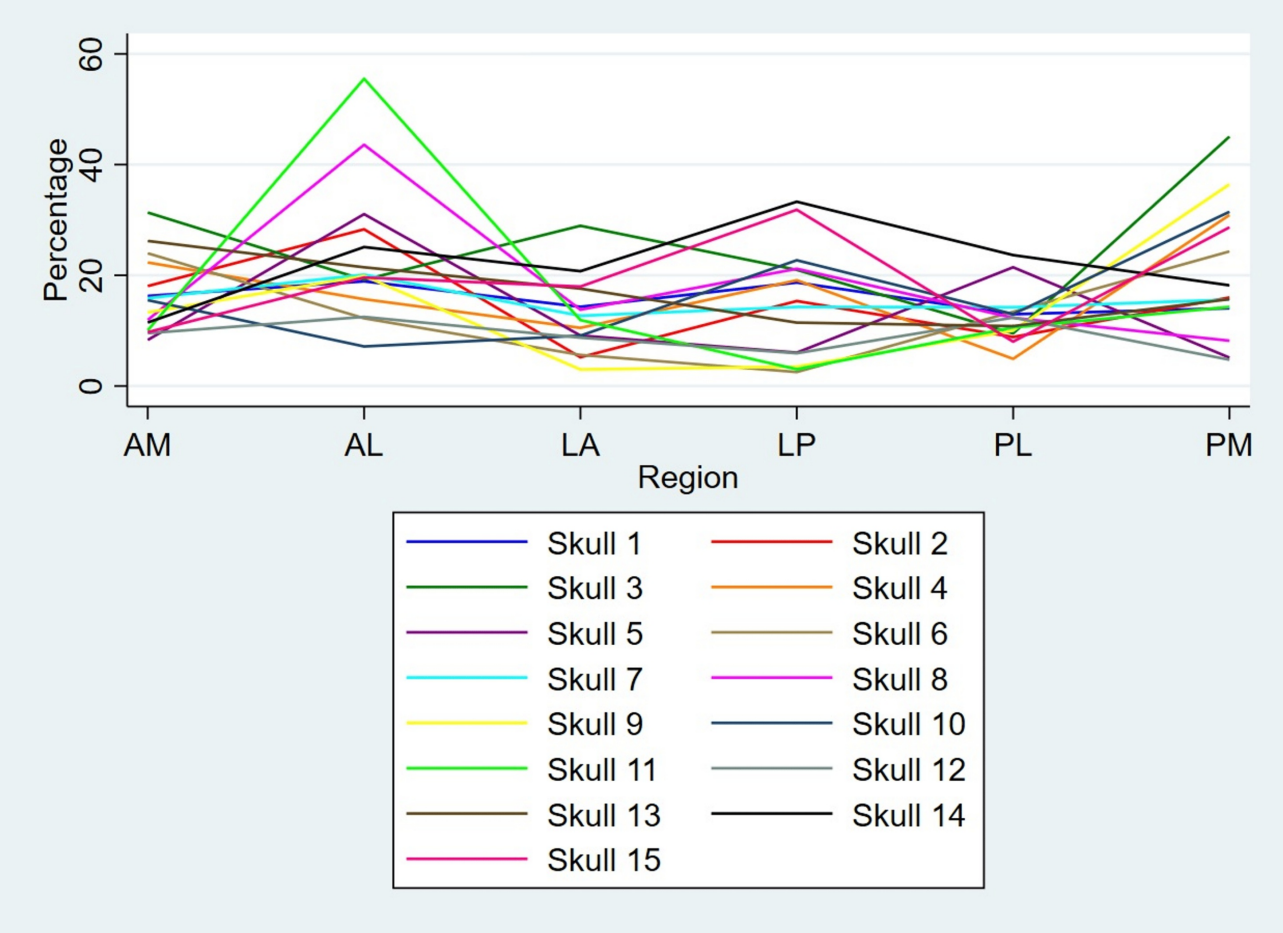
Coefficient of variation (expressed as percentage) in six regions along the left side Coefficient of variation (expressed as percentage) in six regions along the left side of skull margin among the 15 skulls. Skull 8 and 11 show high variation in anterolateral (AL) region, and skull 3 shows high variation in posteromedial (PM) region. (“High variation” implies “>40%”)

**Figure 9:**
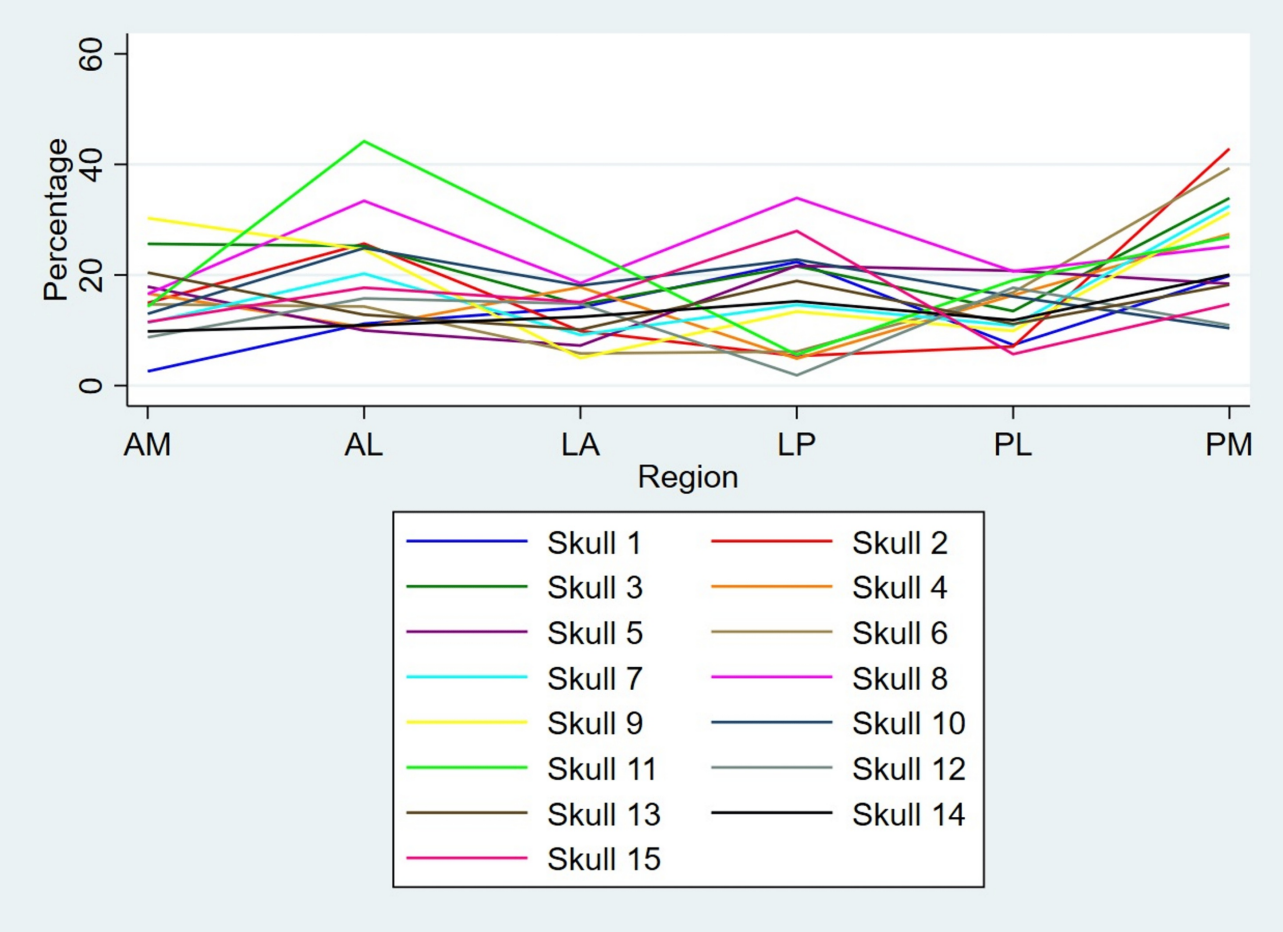
Coefficient of variation (expressed as percentage) in six regions along the right side Coefficient of variation (expressed as percentage) in six regions along the right side of skull margin among the 15 skulls. Skull 11 shows high variation in anterolateral (AL) region, and skull 2 shows high variation in posteromedial (PM) region. (“High variation” implies “>40%”)

Coefficient of variation (expressed as %) in thickness in six regions of the skull margin on both left and right sides in all 15 skulls is presented in Figures 8, 9, respectively.

Two skulls showed high variation in the AL region on the left side. Among these two, one skull showed a high variation (>40%) in the AL region on the right side as well. In the PM region, one skull each showed high variation on the left and right sides, respectively.

Correlation between circumference, midline, and regional thickness

A positive correlation was observed between circumference and PL thickness on the left side (0.5196). Posterior midline showed a positive correlation with PM region on both the left and right sides [left (0.8389)>right (0.5713)], and AL region on the left side (0.5148). Anterior midline showed, on the right side, a positive and a negative correlation with the AM region (0.4809) and the PM region (-0.4922) respectively (Table 3).

**Table 3:** Correlation coefficients between circumference, midline thickness, and regional thickness It shows the correlation coefficients between circumference, midline thickness, and regional thickness of the left and right sides of the skull margins. Superscript ‘*’ marked values indicate a strong correlation.

	Right			Left		
	Circumference	Posterior midline	Anterior midline	Circumference	Posterior midline	Anterior midline
Posterior midline	-0.0258	1		-0.0258	1	
Anterior midline	-0.0838	-0.1032	1	-0.0838	-0.1032	1
AM	-0.1116	0.0823	0.4809	-0.0240	-0.0229	0.4022
AL	-0.1443	0.4579	0.0668	-0.2819	0.5148*	0.0270
LA	0.1977	0.3054	0.0783	-0.0552	0.3542	-0.0038
LP	0.2990	0.0354	-0.0483	0.3563	0.0389	0.0012
PL	0.4528	0.0380	0.1967	0.5196*	0.1804	0.2402
PM	0.1427	0.5713*	-0.4922	-0.0199	0.8389*	-0.1838

Interobserver variation

Since the thickness at each point was measured by three observers, the interobserver variation was expressed using the intraclass correlation coefficient (ICC). More than 80% of measurements showed an ICC value >0.9, thus confirming the high consistency and reliability of the measurements (Table 4).

**Table 4:** Intraclass Correlation Coefficient (ICC) to assess the consistency among 3 observers. The table shows the ICC to assess the consistency among three observers. ICC>0.9 indicates high consistency and reliability. The table also mentions the 95% confidence interval for each ICC. CI: confidence interval.

Region	ICC (Left)	95% CI (Left)	ICC (Right)	95% CI (Right)	Remarks
AM	0.982	0.959–0.993	0.980	0.957–0.991	Excellent
PM	0.976	0.946–0.991	0.987	0.972–0.994	Excellent
skull_1	0.972	0.942–0.988	0.980	0.957–0.991	Excellent
skull_2	0.951	0.899–0.980	0.831	0.676–0.926	* Moderate
skull_3	0.956	0.909–0.982	0.877	0.757–0.947	* Good
skull_4	0.904	0.806–0.959	0.893	0.785–0.954	* Good
skull_5	0.913	0.823–0.963	0.954	0.905–0.981	Excellent
skull_6	0.919	0.835–0.966	0.958	0.912–0.982	Excellent
skull_7	0.983	0.963–0.993	0.974	0.945–0.989	Excellent
skull_8	0.975	0.948–0.990	0.987	0.972–0.994	Excellent
skull_9	0.903	0.804–0.959	0.897	0.793–0.956	* Good
skull_10	0.964	0.925–0.985	0.965	0.926–0.985	Excellent
skull_11	0.977	0.952–0.990	0.968	0.932–0.986	Excellent
skull_12	0.944	0.883–0.976	0.835	0.684–0.928	* Moderate
skull_13	0.917	0.831–0.965	0.897	0.794–0.956	* Good
skull_14	0.919	0.835–0.966	0.969	0.935–0.987	Excellent
skull_15	0.971	0.938–0.988	0.973	0.943–0.989	Excellent

## Discussion

Sectioning of the cranium is indispensable for exposing the meninges and brain during the teaching of neuroanatomy. Motorised circular saws are generally preferred over manual methods because they are faster and produce clean cuts without bony spicules, which may injure the operator. However, improper control of cutting depth can inadvertently damage the meninges and brain. This gap in anatomical knowledge formed the basis of our study.

Traditionally, skull thickness has been assessed either radiographically or directly on dry bones. In a comparative study, 500 lateral view routine radiographs were obtained in a population of two different races (viz. R1 and R2) in the United States, and the thickness was measured at 3 cm anterior (A) and posterior (B) to the coronal suture, and 3 cm superior (C) and inferior (D) to lambdoid suture. Among both females and males, at point A, the values for R2 were greater than R1, and at points C and D, the values for R1 were greater than R2 [7]. In another study, X-rays of 111 skulls excavated in Israel and Jordan were examined, and the thickness was measured at three points in the cranial vault. A mean thickness of 7.9 mm at lambda, 5.6 mm at vertex, and 5.4 mm at bregma was reported [8]. These three points lie in the midline and above the plane of sectioning described in our study. The minimum thickness in the anterior midline reported in our study is 7.409 mm. Thus, from bregma downwards, the thickness increases. This can be attributed to the internal frontal crest. The posterior midline showed a thickness ranging from 5.821 mm to 14.068 mm. Such a wide range in thickness can be due to variability in the position of the internal occipital protuberance, which may (or may not) be situated exactly opposite the external occipital protuberance. Therefore, from lambda downwards, the thickness may decrease by a couple of millimeters or increase by up to two times. In modern and neolithic populations in Japan, thickness at frontal and parietal eminences were significantly greater in women than in men [9]. The measurement of 15 points on 88 parietal bones from 44 Korean adult skulls showed the posteromedial part of the bone to be the thickest (6.67±1.41 mm) and anterolateral part to be the thinnest (4.73±1.19 mm) [10]. Our study also established the PM region to be the thickest; however, this region in the skull margin is formed mostly by the occipital bone. The LA region (minimum mean thickness) described in our study is formed mostly by the AL part of the parietal bone, which has already been described as the thinnest part in the aforementioned study. In a study involving four cranial segments from the frontal and parietal regions of 165 specimens collected at autopsy and 15 calvaria specimens, an increase in cranial thickness with age was observed, and cranial thickness was not found to be sexually dimorphic [11]. Another study involving 40 points on the calvaria in 281 dry skulls showed a mean thickness of 6.32 mm and reported the parasagittal posterior parietal area to be the thickest [12]. This posterior parasagittal area roughly corresponds to the PM region described in our study, and therefore our study reaffirms the fact. However, most of the points among the 40 points selected in this study are placed on the vault of the calvaria. In contrast to previous studies, the current study involved measurement of points along a lower and transverse plane. The previous studies reported thickness values up to one/ two decimal places, but we have been successful in reporting them more precisely up to three decimal places.

To the best of our knowledge, this study is the first to introduce a six-region classification (Figure 3) of the skull margin applicable to neurosurgical, radiological, forensic, and educational practice. These regions from anterior to posterior are as follows: anteromedial (AM), anterolateral (AL), lateral anterior (LA), lateral posterior (LP), posterolateral (PL), and posteromedial (PM). The regional thickness on both sides decreased from AM to LA region and then increased up to PM region (Figure 7). Thus, the thinnest and thickest margins were observed in the LA and PM regions, respectively. Hence, an equal amount of force can result in greater damage in the LA region compared to the PM region. In neurosurgery, thin regions such as LA require careful drilling depth control while performing a burr hole craniotomy and during halo pin fixation to prevent dural injury. On the contrary, thick PM region may be optimal for harvesting cranial bone for an autologous graft in cases of facial/ orbital/ temporal fractures and also in cases of large cranial defects. The maximum mean thickness was more than (or nearly) twice the minimum mean thickness in the anterior midline, posterior midline, and PM region (Figure 7). The reason for such a wide range in thickness requires further exploration and underscores the need for region-specific surgical caution.

In radiology, cross-sectional imaging (CT and MRI) is routinely used for trauma and intracranial pathology assessment. The six-region classification can refine lesion localization (such as skull fracture, extradural hemorrhage, subdural hemorrhage, subarachnoid hemorrhage, penetrating trauma, superficial cerebrovascular injury, and traumatic brain injury), description, and interpretation of trauma patterns, while also enhancing emerging automated MRI-based skull thickness mapping tools, such as BrainCalculator, as precision instruments for neuromodulation planning and radiological navigation, highlighting the importance of detailed morphometric data [13]. Regional morphometric data can be utilized in anthropometric studies, and can also improve reconstruction accuracy in blunt and ballistic cranial trauma analysis in forensic applications. Moreover, CT morphometric studies demonstrate cortical thinning patterns with age and sex, underscoring the forensic value of region-specific skull thickness data in trauma reconstruction and identification [14]. The present study does not provide any insights into the developmental factors contributing to the diversity in cranial thickness. Additionally, the research lacks information regarding the age and gender of the skull specimens that were examined. The small sample size (n=15) reduces the statistical power and limits the generalization of the findings. Further multicentric studies with larger, more diverse populations and thickness measured in a greater number of parallel planes in horizontal, coronal, and sagittal orientations may be carried out to enhance the utility of such morphometric studies, followed by CT/MRI-based validation.

## Conclusions

The findings of the present study will be useful to human anatomists for sectioning the cadaveric cranium to expose the meninges and the brain for neuroanatomy teaching because the current anatomical literature lacks information about the safe cutting depth. In the anterior and posterior midline, the blade can safely go up to 7 mm and 5 mm, respectively. Readers can refer to Figure 7 for the minimum average thickness in each region along the margin of the skull prior to sectioning the cranium. These data support safer, cleaner motorized saw sectioning, reducing risk to personnel and structures. The six-region classification may be adopted in anatomy and radiology teaching practices.
